# Supplementary material for: The health status alters the pituitary function and reproduction of mice in a Cxcr2-dependent manner
Source: Life Sci Alliance. 2020 Feb 10;3(3):e201900599. doi: 10.26508/lsa.201900599 (PMC7010316; doi:10.26508/lsa.201900599)
Supplement: Supplementary file 1 [file LSA-2019-00599_TableS1.docx]

Supplemental Table 1

| **Signature** | **HGNC symbols** | **Gene Name** | **%** |
| --- | --- | --- | --- |
| B cells | BLK | BLK proto-oncogene, Src family tyrosine kinase | 5/37 genes |
| B cells | CD72 | CD72 molecule |  |
| B cells | CD79A | CD79a molecule, immunoglobulin-associated alpha |  |
| B cells | LOC100507616 | uncharacterized LOC100507616 |  |
| B cells | SNX22 | sorting nexin 22 |  |
| T cells | AMICA1 | adhesion molecule, interacts with CXADR antigen 1 | 18/85 genes |
| T cells | ARHGAP9 | Rho GTPase activating protein 9 |  |
| T cells | BIN2 | bridging integrator 2 |  |
| T cells | BTK | Bruton agammaglobulinemia tyrosine kinase |  |
| T cells | CCL19 | chemokine (C-C motif) ligand 19 |  |
| T cells | CD27 | CD27 molecule |  |
| T cells | CD3D | CD3d molecule, delta (CD3-TCR complex) |  |
| T cells | CD3E | CD3e molecule, epsilon (CD3-TCR complex) |  |
| T cells | CD3G | CD3g molecule, gamma (CD3-TCR complex) |  |
| T cells | CORO1A | coronin, actin binding protein, 1A |  |
| T cells | CXCR6 | chemokine (C-X-C motif) receptor 6 |  |
| T cells | DPEP2 | dipeptidase 2 |  |
| T cells | HMHA1 | histocompatibility (minor) HA-1 |  |
| T cells | ICOS | inducible T-cell co-stimulator |  |
| T cells | PSTPIP1 | proline-serine-threonine phosphatase interacting protein 1 |  |
| T cells | PTPRCAP | protein tyrosine phosphatase, receptor type, C-associated protein |  |
| T cells | SLA | Src-like-adaptor |  |
| T cells | TBC1D10C | TBC1 domain family, member 10C |  |
| Macrophages | CCR1 | chemokine (C-C motif) receptor 1 | 10/78 genes |
| Macrophages | CYTH4 | cytohesin 4 |  |
| Macrophages | IGSF6 | immunoglobulin superfamily, member 6 |  |
| Macrophages | ITGAM | integrin, alpha M (complement component 3 receptor 3 subunit) |  |
| Macrophages | ITGAX | integrin, alpha X (complement component 3 receptor 4 subunit) |  |
| Macrophages | LAPTM5 | lysosomal protein transmembrane 5 |  |
| Macrophages | MS4A4A | membrane-spanning 4-domains, subfamily A, member 4A |  |
| Macrophages | SPI1 | Spi-1 proto-oncogene |  |
| Macrophages | TRPV2 | transient receptor potential cation channel, subfamily V, member 2 |  |
| Macrophages | TYMP | thymidine phosphorylase |  |
| Neutrophils | NCF4 | neutrophil cytosolic factor 4, 40kDa | 3/47 genes |
| Neutrophils | S100A8 | S100 calcium binding protein A8 |  |
| Neutrophils | S100A9 | S100 calcium binding protein A9 |  |
