# Supplementary material for: The health status alters the pituitary function and reproduction of mice in a Cxcr2-dependent manner
Source: Life Sci Alliance. 2020 Feb 10;3(3):e201900599. doi: 10.26508/lsa.201900599 (PMC7010316; doi:10.26508/lsa.201900599)
Supplement: Supplementary file 2 [file LSA-2019-00599_TableS2.docx]

Supplemental Table 2.

| **Gene** | **Upper primer sequence** | **Lower primer sequence** |
| --- | --- | --- |
| **Gapdh** | GGTGCTGAGTATGTCGTGGA | GTGGTTCACACCCATCACAA |
| **Rs9** | CGGCCCGGGAGCTGTTGACG | CTGCTTGCGGACCCTAATGTCACG |
| **Cxcr2** | CTCATCACGGCTGCCTCACTTTC | AATGGCCAGGTTCAGCAGGTAGAC |
| **S100a8** | CAATGCCGTCTGAACTGGAG | AAGCTCTGCTACTCCTTGTGG |
| **S100a9** | TGGGCTTACACTGCTCTTACC | GGTTATGCTGCGCTCCATCT |
| **Mmmp8** | CCTTGCCCATGCCTTTCAAC | TCATGAGCAGCCACGAGAAA |
| **Ngp** | ATACAACCAAGGGCGGCAAG | ATCCTCCAGGAAGTCGCAGT |
| **Areg** | CCCGGAGCCGCTGTCGTGTT | GCCAGCGGTAGCAGCGGAGTTCT |
| **Lactoferrin** | GCGGGCAAGTGCGGTTTAGTT | CACAGGGCACAGAGATTGGATTTG |
| **Cxcl15** | GGCATCTTCGTCCGTCCCTGTG | CAGTAGCCTTCACCCATGGAGCA |
| **Elf5** | GTCAAGACTGTCACAGCCGA | TTCCCATTCCAGGATGCCAC |
| **Sox10** | GAAGAAGGCTCCCCCATGTC | TTGGGTGGCAGGTATTGGTC |
| **Ido1** | GGCTAGAAATCTGCCTGTGC | AGAGCTCGCAGTAGGGAACA |
| **Wnt2** | ACCTGATGTAGACGCAAGGG | CCAATGGCACGCATCACATC |
| **Prlr** | ACCTGTCACTGAAAAAGCTCGCGTT | TGTTTTGCTTCAGGCTGGCCCT |
| **Krt15** | CTGCCACCATTGACAACTCG | TGCTGCGTCCATTTCCACAT |
| **Gata3** | TCCGTCAGGGCTACGGTGCA | GACGTCTTTCGGCGGGGTGG |
| **Akr1c18** | GTCCTGGGCTTTGGCACCTATGC | GGGTCTGACCAACTCTGGACGA |
| **Cyp19** | TTGGACCGGCTGGGCGAAGT | ATGAGGAGAGCTTGCCAGGCGT |
| **Hsd3b2** | CGGGCCCAACTCCTACAAG | TTTTCCAGAGGCTCTTCTTCGT |
| **Ar** | AGGTCTTCCCCTGGACGAA | TTTGGCCTAACCTCCCTTGA |
| **Elane** | CATGGCCCTTGGCAGACTAT | AGTTCCTGGCAATGAGGGTG |
| **Mpo** | GAACAATCAGTACCGGCCCA | GAGCAGGCAAATCCAGTCCT |
| **Serpina3an** | CTGGCTGGTTTCAGCTCTGT | ATCCATTCCCAACGTGCCAT |
| **Prl** | CTGCTGCTAGGCTTAGTCCTC | AGGCAGGATTGATGTCAGGG |
| **Crhbp** | TGTGTTACCCCTTTCTCGGC | GGCTCCAGCTGACGATACTC |
| **Akr1c14** | CCACTGTGCCCGATAAGGTT | GGTCGATGGAAAGTGCTCCA |
| **Vip** | CAGGTGACCCTGACCAAGTC | AGATGCTGCTGCTGATTCGT |
| **Reln** | TCAGCGGGTGTCTTACAACG | TTTGCGAGTGCTTACTAGGAC |
